# Mycotoxins occurrence in medicinal herbs dietary supplements and exposure assessment

**DOI:** 10.1007/s13197-021-05306-y

**Published:** 2021-11-10

**Authors:** Noelia Pallarés, Houda Berrada, Guillermina Font, Emilia Ferrer

**Affiliations:** Laboratory of Toxicology and Food Chemistry, University of Valencia, Avda. Vicent Andrés Estellés s/n, 46100 Burjassot (Valencia), Spain

**Keywords:** Multimycotoxin, Medicinal herbs, QuEChERS, LC–MS/MS-IT, Dietary exposure

## Abstract

The multimycotoxin analysis of aflatoxins (AFs), zearalenone (ZEA), ochratoxin A (OTA), enniatins (ENNs) and beauvericin (BEA) was performed in 85 samples of medicinal herbs dietary supplements. The samples were classified in 64 samples of one herbal ingredient and 21 mixed samples. The extraction was performed by QuEChERS method and the determination by liquid chromatography coupled to ion-trap tandem mass spectrometry (LC–MS/MS-IT). Then, the risk characterization to mycotoxins through the consumption of medicinal herbs dietary supplements was assessed. The results showed that ZEA, OTA, ENNs and BEA showed in the samples with incidences between 1 and 34%, being ENNB the most detected mycotoxin. Mycotoxins contents ranged from LOQ to 3850.5 µg/kg while the mean of positives samples were 65.5 µg/kg (ENNA), 82.7 µg/kg (ENNA1), 88.7 µg/kg (ENNB), 324.9 µg/kg (ENNB1), 137.9 µg/kg (BEA) and 1340.11 µg/kg (ZEA), respectively. OTA was detected in one herbal mix tablet for insomnia at concentration of 799 μg/kg. In herbal drugs the European Pharmacopoeia Commission has implemented limits of 2 µg/kg for AFB1 and 4 µg/kg for total AFs. In the present study AFs have not been detected in the analyzed medicinal herbs dietary supplements. The Estimated Daily Intakes (EDIs) values were calculated using a deterministic method, considering two exposure scenarios (lower bound (LB) and upper bound (UB)). The values obtained were in general far below the Tolerable Daily Intakes (TDIs) established.

**Graphical abstract:**

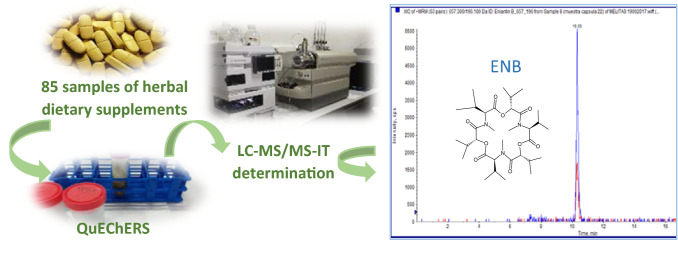

**Supplementary Information:**

The online version contains supplementary material available at 10.1007/s13197-021-05306-y.

## Introduction

The use of medicinal herbs to improve quality of life is a practice expanded all over the world, not only in developing countries where high percentage of population depend upon medicinal herbs as a primary health care source, but also in highly developed countries, due to the self-medicate tendency and the growing population acceptance of natural products (Abdel-Tawab 2018). These botanicals are available in the corresponding markets in several forms: plant food supplements, homeopathic products, foods (teas and juices), and cosmetic products. They are commonly used to treat some acute and chronic neck pain, colds, anxiety, or depression (Abdel-Tawab 2018; Hudson et al. 2018).

The World Health Organization (WHO) has estimated a demand growing for medicinal herbs and their products at the rate of 15 to 25% annually and it is expected that by 2050 the trade will be up to US$ 5 Trillion. Medicinal herbs are cultivated in different regions around the world, being China and India the main producers. In the European Union (EU), a cumulative area of 70,000 ha is used to cultivate medicinal and aromatic plants (Asiminicesei et al. 2020). The imports of medicinal herbs in the EU were estimated in 116.3 thousand tones, while the exports corresponded to 41.9 thousand tones in 2002, being the EU one of the Organization for Economic Co-operation and Development (OCED) regions most involved in medicinal herbs trade, with imports figures greater than exports. While China and India are the top exporting countries, Hong Kong, Japan, USA and Germany constitute the leading importers (FAO 2005). In Europe, the turnover in medicinal herbs is reported with a retail sales volume estimated in $ 6 billion per annum (Haq 2004).

Due to tremendous demand increasing for medicinal herbs, there is an urgent need to guarantee the standardization of herbal products and the lack of toxicity that may be caused by heavy metals, pesticides, microbial contaminants, and chemical toxins. These toxic substances are produced under unfavorable or inadequate storage conditions (Nirmal et al. 2013).

Some adverse effects related to medicinal herbs are hepatotoxicity, cardiovascular toxicity, and central nervous system alterations related to toxic effects of active plant compounds or to supplement’s contamination during manufacturing process and the improper use (Hudson et al. 2018).

The increased popularity of medicinal herbs has forced the introduction of several regulations all over the world to guarantee public health and to assure their quality, efficacy, and safety (Qu et al. 2018).

In the EU, from the legal point of view, food supplements are considered as food. There is no special category for these products that imply any safety assessment, prior to their placement in the market. Furthermore, the frame legal of supplements is not totally harmonized, and a substance or product that is considered food supplement in a European country may not be considered in another European country (Troncoso 2019).

For herbal dietary supplements, the EU monograph provides a system for the regulation together with the European Pharmacopeia which defined basic quality requirements for herbal medicinal products. The EU monograph is established by the Committee on Herbal Medicinal Products (HMPC), that was constituted as one of the scientific committees of the European Medicines Agency under the regulation 2004/24/EC, which amends Directive 2001/83/EC.

According to 2004/24/EC, medicinal herb products must provide all information for market authorization, with exception of preclinical and clinical data. The efficacy and safety can be demonstrated by HMPC monographs (Abdel-Tawab 2018).

Major contaminants of medicinal herbs and products vary from heavy metals, pesticide residues to mycotoxins (Kosalec et al. 2009). Regarding mycotoxins, medicinal herbs supplements can be contaminated by various toxigenic fungi during harvesting, handling, storage, and distribution. The risk of contamination by mycotoxigenic fungi and subsequently with mycotoxins, increase with poor agricultural and harvesting practices or inadequate conditions of storage, distribution, or transportation (Ashiq et al. 2014). Poor qualities of raw materials can also affect the final product. Mycotoxins are consequence of fungal growth; thus, its presence indicates hygienic deficits during production and storage (Ałtyn and Twaru˙zek 2020). In general, medicinal herbs are produced through the traditional open, small workshop, and scattered planting business models. The lack of an uniform standard or efficient supervision method may damage them during processing, storage, and transportation steps. The intrinsic factors join to external environmental conditions lead to severe spoilage and deterioration, along with mycotoxigenic fungi contamination (Chen et al. 2020). *Aspergillus* and *Penicillium* constitute the predominant genera in medicinal herbs with capacity to produce mycotoxins. Several environmental factors such as temperature or relative humidity are reported to be those that most influence mycotoxins production (Alwakeel 2009).

Mycotoxins are natural toxicants that are produced by a high number of species belonging to different fungal generas, mainly to *Fusarium*, *Claviceps*, *Alternaria*, *Aspergillus* and *Penicillium*. These compounds are related to adverse carcinogenic, genotoxic, teratogenic, dermatotoxic, nephrotoxic and hepatotoxic effects in animals and humans (Marin et al. 2013).

Most methods employed for mycotoxins extraction from food matrixes involve sample pretreatments, homogenization, and cleanup strategies. Those extraction methods, such as liquid–liquid extraction (LLE), supercritical fluid extraction (SFE), solid phase extraction (SPE), immunoaffinity column clean-up (IAC) and dilute and shoot enhance recoveries and efficiency. Moreover, analytical techniques such as gas chromatography (GC), liquid chromatography (LC), mass spectrometry (MS), capillary electrophoresis (CE) and thin-layer chromatography (TLC) have been employed for mycotoxins identification and quantification. LC and GC coupled to MS are the most selective and sensitive used ones (Zhang and Banerjee 2020; Qin et al. 2020).

As far as mycotoxins are concerned, the European Pharmacopoeia Commission has implemented stricter limits for the presence of Aflatoxins (AFs) in herbal drugs limit set to 2 µg/kg for aflatoxin B1 (AFB_1_) and to 4 µg/kg for total aflatoxins (European Pharmacopoeia, 2016). However, other mycotoxins such as emerging mycotoxins have not been regulated yet. There is an urgent need for the creation or updated legislation to cover traditional mycotoxins as well as emerging mycotoxins such as enniatins (ENNs) and beauvericin (BEA) and masked mycotoxins. Only in this way, medicinal herbs will meet the precepts of food safety.

Information about mycotoxin contamination in various types of medicinal herbs dietary supplements is scarce (Veprikova et al. 2015), focusing in one ingredient, such as green tea supplements (Martínez-Domínguez et al. 2016), milk thistle supplements (Arroyo-Manzanares et al. 2013) or ginkgo biloba supplements (Di Mavungu et al. 2009; Martínez-Domínguez et al. 2015).

In this context, the aim of the present study was to perform a multimycotoxin analysis (AFs, Zearalenone (ZEA), Ochratoxin A (OTA), ENNs and BEA) in 64 tablet samples of medicinal herbs dietary supplements containing one herbal ingredient, and 21 mix tablet samples. The extraction was performed by QuEChERS method and the determination by liquid chromatography coupled to ion-trap tandem mass spectrometry (LC–MS/MS-IT). An estimation of the population's risk to mycotoxins through the intake of medicinal herbs dietary supplements was also performed.

## Material and methods

### Reagents and chemicals

Acetonitrile (ACN) and methanol (MeOH) HPLC grade were purchased from Merck (Darmstadt, Germany). Deionized water (resistivity > 18 MΩ cm^−1^) was obtained using a Milli-Q SP® Reagent Water System (Millipore Corporation Bedford, USA). Ammonium formate (99%) was supplied by Panreac Quimica S.A.U. (Barcelona, Spain) and formic acid (reagent grade ≥ 95%) was supplied by Sigma-Aldrich (St. Louis, MO, USA). All solvents were filtered through a 0.45 μm cellulose filter supplied by Scharlau (Barcelona, Spain) before use. Salts for QuEChERS extraction: sodium chloride (NaCl) was obtained from VWR Chemicals (Leuven, Belgium), Magnesium sulfate (MgSO_4_), anhydrous 99.5% min powder was supplied by Alfa Aesar (Karlsruhe, Germany) and Octadecyl C18 sorbent was acquired from Phenomenex (Madrid, Spain). Before injection, samples were filtered through a nylon filter (13 mm/0.22 μm) from Membrane Solutions (TX, USA). Mycotoxins standards (AFB_1_, AFB_2_, AFG_1_, AFG_2_, ZEA, OTA, ENNA, ENNA_1_, ENNB, ENNB_1_ and BEA) were supplied by Sigma (St. Louis, MO, USA). Individual stock solutions of each mycotoxin were prepared in MeOH at concentration of 100 mg/l. The working solutions were prepared from these individual stock solutions. All prepared solutions were stored in darkness at − 20 °C until the analysis.

### Sample collection

85 tablet samples of the most common medicinal herbs dietary supplements used as natural remedies in Spain were acquired from different herbalists or pharmacies located in Valencia (Spain). These samples were 64 tablets based on one herbal ingredient (horsetail “*Equisetum arvense L.*”, artichoke “*Cynara scolymus*”, valerian root “*Valeriana officinalis*”, dandelion plant “*Taraxacum officinale*”, *cardus marianus* “*Silybum marianum*”, fucus “*Fucus vesiculosus L*.”, boldus leaves “*Peumus boldus”*, ginkgo “*Ginkgo biloba”*, ginger “*Zingiber officinale”*, passionflower “*Passiflora incarnata*”, devil's clawroot “*Harpagophytum procumbens”*, whitethorn “*Crataegus monogyna”*, lemon balm leaves “*Melissa officinalis”*, red tea “Aspalathus linearis” and green tea *“Camellia sinensis*”), acquiring at least four samples for each herbal type, and 21 samples that are based on more than one herbal ingredient (Table S1), which are used to treat insomnia or to lose weight. Samples were stored in their original packaging in a dark and dry place until the analysis. Table S1 describes the botanical contents of the analyzed tablets, the dosage recommended by the manufacturer and the main health effects associated.

### QuEChERS procedure extraction

The analysis was performed using a QuEChERS procedure for mycotoxin extraction and the method was in-house validated. The tablets were crushed and 2 g of their content were weighted in a 50 ml falcon tube before adding 10 ml of acidified water with 2% formic acid and shacked for 30 min in a shaker KS 260 IKA (Staufen, Germany) at 200 rpm. Then, 10 ml of ACN were added and the resulting mixture was shacked for other 30 min using the same shaker. Then, 4 g of MgSO_4_ and 1 g of NaCl salts were added to the tube and the mixture was vortexed for 30 s and centrifuged in a Centrifuge 5810 R Eppendorf (Madrid, Spain) at 5000 rpm during 10 min. 2 ml of the supernatant were taken and placed into a 15 ml falcon tube with 0.3 g of MgSO_4_ and 0.1 g of Octadecyl C18 sorbent, then the mixture was shacked again with the IKA shaker and after was centrifuged at 5000 rpm for 10 min. The obtained supernatant was filtered with a 13 mm/0.22 μm nylon filter (Membrane Solutions, TX, USA), prior to injection of 20 μl into the LC–MS/MS-IT system.

### LC–MS/MS-IT analysis

An Agilent 1200 chromatograph (Agilent Technologies, Palo Alto, CA, USA) equipped with 3200 QTRAP® (Applied Biosystems, AB Sciex, Foster City, CA, USA) with Turbo Ion Spray (ESI) electrospray ionization was used for the determination. The QTRAP analyser combines a fully functional triple quadrupole and a linear ion trap mass spectrometer. The column for the analyte separation was a Gemini-NX column C_18_ (Phenomenex, 150 mm × 4.6 mm, 5 particle size) preceded by a guard column. The flow rate was set at 0.25 ml/min, and the oven temperature at 40 °C. The elution mobile phases consisted in acidified water with 5 mM ammonium formate and 0.1% formic acid (mobile phase A) and in acidified methanol with 5 mM ammonium formate and 0.1% formic acid (mobile phase B). For the elution, the gradient started with 0% of eluent B; in 10 min increased to 100%, decreased to 80% in 5 min, and to 70% in 2 min. In the next 6 min, the column was readjusted to initial conditions and equilibrated for 7 min.

The Turbo Ion Spray was used in positive ionization mode (ESI +). Nitrogen was served as nebulizer and collision gas. The conditions employed were: Ion spray voltage 5500 V; curtain gas 20 arbitrary units; GS1 and GS2 with 50 and 50 psi, respectively and probe temperature (TEM) of 450 C.

The quantification and confirmation transitions of mycotoxin monitored fragments and the spectrometric parameters (declustering potential, collision energy and cell exit potential) are shown in Table S2.

### Method optimization

The method was optimized for medicinal herbs dietary tablets in terms of recoveries, repeatability (intra-day precision), reproducibility (inter-day precision), matrix effects (signal suppression-enhancer), linearity, and limits of detection (LODs) and quantification (LOQs) according to Commission Decision (2002/657/EC). The analytical parameters are shown in Table S3.

Recoveries were determined by spiking blank horsetail tablet samples with each studied mycotoxin at 100 × LOQ concentration level, before and after the QuEChERS extraction in triplicate. To assess the intra-day precision, three determinations were performed on the same day and on nonconsecutive days to assess the inter-day precision. Intra-day and inter-day recoveries obtained ranged from 73 to 117% and were within the relative standard deviation (< 20%).

Matrix effects (MEs) values, calculated to evaluate a possible suppression or enhancement of the original signal (SSE), were obtained comparing the slope of the calibration curve prepared in blank horsetail tablet samples with the slope of the calibration curve prepared in methanol. SSE (%) were calculated as follows: SSE(%) = 100 × slope with matrix/slope without matrix. Signal Suppression-Enhancer (SSE) for matrix effects were between 46 to 98%, observing the highest suppression ME for AFs. To minimize MEs, analytical parameters were determined using matrix matched calibration curves.

The LODs and LOQs were calculated by spiking a blank horsetail tablet samples with decreasing concentrations of the analyzed mycotoxins using the criterion of S/N ≥ 3 for calculating the LOD and S/N ≥ 10 for the LOQ. The LODs and LOQs ranged from 0.15 to 3 µg/kg and from 0.5 to 10 µg/kg, respectively.

Calibration curves in both pure solvent (methanol) and blank horsetail tablet samples were constructed at eight concentration levels (from LOQs to 1000 µg/kg). Linearity (r2) was in the range from 0.991 to 0.999 for all studied mycotoxins. Therefore, matrix-matched calibration curves constructed by spiking blank horsetail tablet samples were used for effective quantification of samples.

### Risk assessment

A deterministic approach was performed for risk assessment. For this purpose, the Estimate Daily Intakes (EDIs) to mycotoxins were calculated and compared with their Tolerable Daily Intakes (TDIs).

EDI for each mycotoxin was calculated with the combination of supplement medium recommended dosage and mycotoxins mean concentration in each type of supplements, considering a medium corporal weight of 70 kg. The medium recommended dosage was calculated according to the information of recommended dosage supplied by the different manufacturers per each type of herbal dietary supplement. Therefore, EDI (µg/Kg bw/day) was obtained as follows = recommended dosage daily consumption (g/kg bw/day) × medium concentration of each mycotoxin in each type of herbal dietary supplement (µg/g).

The medium concentration for detected mycotoxins has been calculated considering two scenarios, lower bound (LB) and upper bound (UB). In LB, a value of 0 was assigned to samples where mycotoxins were not detected or were detected at concentrations below LOQ. In UB, the values of LODs were assigned to samples where mycotoxins were not detected, and the values of LOQs were assigned to samples where mycotoxins were detected at concentrations between LOD and LOQ (EFSA 2010).

According to the safety guidelines, a TDI of 0.25 µg/kg bw/day has been fixed for ZEA (EFSA 2014) and a Tolerable Weekly Intake (TWI) of 0.12 µg/kg bw/week for OTA (EFSA 2006). For emerging mycotoxins, TDI values have not yet been fixed, but the EDIs obtained in the present work can be compared with the lowest and highest TDI values fixed for other Fusarium mycotoxins, DON (1 μg/kg bw/day)(SCF 2002) and the sum of the toxins HT2 and T2 (0.1 μg/kg bw/day) (EFSA 2014).

### Statistical section

Student’s *t*-test statistical analysis was carried on assessing significant differences between ecological and conventional supplements. *P*-values of < 0.05 were considered to be statistically significant. Data was expressed as mean ± standard error of the mean.

## Results and discussion

### Mycotoxin occurrence in medicinal herbs dietary tablets

All studied mycotoxins were detected in the analyzed samples except AFs. ENNB was the most detected mycotoxin (34%), followed by ENNA_1_ (14%) and ENNB_1_ (13%) (Fig. S1). Although ENNB presented the highest incidence, the corresponding mean concentration of positive samples quantified (88.7 μg/kg) located in the bottom of the detected mycotoxins. ZEA showed up at 8% of tablet samples but presented the highest mean concentration (1340.1 μg/kg). OTA was detected in one herbal mix tablet for insomnia but at a considerable concentration (799 μg/kg). BEA was detected in 11% of analyzed tablets, with mean concentration of 137.89 μg/kg (Table 1). Figure S2 shows a chromatogram of a whitethorn tablet naturally contaminated by ENNB.

**Table 1:** Minimum, maximum mycotoxin concentrations, mean of positive samples (µg/kg) and incidences of detected in medicinal herbs dietary tablets

Mycotoxin	ZEA	OTA	ENNA	ENNA_1_	ENNB	ENNB_1_	BEA
Minimum Concentration (µg/kg)	116.9	799	3.8	< LOQ	< LOQ	< LOQ	< LOQ
Maximum Concentration (µg/kg)	3850.5	799	170.8	534.9	1378.2	1188.3	542.7
Mean of positive tablets ± SD (µg/kg)	1340.1 ± 1290	799 ± 0	65.5 ± 55	82.7 ± 156	88.7 ± 277	324.9 ± 497	137.9 ± 168
Incidence^a^	7/85	1/85	8/85	12/85	29/85	11/85	9/85

^a^ number of positive samples/ number of total samples

As mentioned above, European Pharmacopoeia recommendation established a maximum level of AFB_1_ as well as total AFs in herbal drugs, however in the present work AFs were not detected in none of the analyzed samples.

### Mycotoxins contents per type of medicinal herb

Artichoke, green tea, red tea, ginkgo tablets showed no mycotoxin contamination in any of the analyzed samples (Table 2). The tablets for lose weight resulted also not contaminated, these tablets were made mainly with green tea and fucus, only one of the five samples of individual fucus tablets resulted contaminated by ZEA at 659.73 μg/kg. In a previous study, Pallarés et al. (2017) did not report either contamination of mycotoxins at levels above the limit of quantification in samples of green and red teas prepared as aqueous infusions. The major part of studies available in literature are focused on specific types of medicinal herbs supplements, like ginkgo biloba, ginseng, *cardus marianus* or green tea. Di Mavungu et al. (2009) did not detect the presence of any mycotoxin in ginkgo. Contrary to these results, Martínez-Domínguez et al. (2015) observed the presence of AFB_1_, AFB_2_, T-2 with incidences of 14, 29 and 29% respectively in seven samples of ginkgo biloba leaves extracts. In multimycotoxin analysis performed in green tea samples, Martínez-Domínguez et al. (2016) only detected AFB_1_ in one of ten samples at 5.4 μg/Kg.

**Table 2:** Mycotoxins contents and incidence per type of medicinal herbs

Type of medicinal herbs tablet (n^a^)	Mycotoxin Concentration range µg/kg and Incidence^b^						
	ZEA	OTA	ENNA	ENNA_1_	ENNB	ENNB_1_	BEA
Artichoke (4)	nd	nd	nd	nd	nd	nd	nd
Boldus (4)	(1169–1995.9)	nd	nd	nd	(1.8–5.4)	(< LOQ)	nd
	(3/4)				(3/4)	(1/4)	
*Cardus Marianus* (4)	nd	nd	(36.6–109.2)	(57.6–534.9)	(6.2–1378.2)	(24.2–1165.9)	(< LOQ-542.7)
			(2/4)	(2/4)	(3/4)	(2/4)	(2/4)
Dandelion (5)	nd	nd	(39.6)	(26.6–28.3)	(4.8–74.6)	(< LOQ-71.5)	nd
			(1/5)	(2/5)	(4/5)	(3/5)	
Devil’s Clawroot (4)	(212.6)	nd	nd	nd	(2.5–2.7)	nd	nd
	(1/4)				(2/4)		
Fucus (5)	(659.7)	nd	nd	nd	nd	nd	nd
	(1/5)						
Ginger (4)	(3850.5)	nd	nd	nd	(3.3–15.1)	nd	(95.7–136.8)
	(1/4)				(2/4)		(3/4)
Ginkgo (4)	nd	nd	nd	nd	nd	nd	nd
Green tea (5)	nd	nd	nd	nd	nd	nd	nd
Horsetail (5)	nd	nd	(12.6–170.8)	(35.2–156.5)	(7.1–588.6)	(377–1188.3)	(25.1–52.2)
			(2/5)	(2/5)	(3/5)	(2/5)	(2/5)
Lemon balm (3)	(117)	nd	nd	nd	(6.6)	nd	nd
	(1/3)				(1/3)		
Passionflower (4)	nd	nd	nd	nd	(3.1–9.1)	nd	(70.2)
					(3/4)		(1/4)
Red tea (4)	nd	nd	nd	nd	nd	nd	nd
Valerian (5)	nd	nd	(63.1–88.7)	(8.5–42.7)	(0.8–27.8)	(22.3)	nd
			(2/5)	(2/5)	(4/5)	(1/5)	
Whitethorn (4)	nd	nd	nd	(6.6–12.3)	(2.3–15)	(10.7–22.2)	(47.2)
				(2/4)	(2/4)	(2/4)	(1/4)
Herbal mix for treat insomnia (16)	nd	(799)	(3.8)	(< LOQ-1)	(< LOQ-1.5)	nd	nd
		(1/16)	(1/16)	(2/16)	(2/16)		
Herbal mix for lose weight (5)	nd	nd	nd	nd	nd	nd	nd

^a^ number of samples ^b^ number of positive samples/ number total samples

#### Mycotoxins contents in positive samples

At least one of the analyzed tablets of valerian, dandelion, boldus, ginger, passionflower, horsetail, *cardus marianus*, devil’s clawroot, whitethorn, lemon balm, fucus and herbal mix used to treat insomnia resulted contaminated by one mycotoxin.

Per type of botanical contents, boldus, *cardus marianus*, horsetail and ginger tablets were the most contaminated tablets. These botanicals presented co-occurrence of mycotoxins at levels up to 2000 µg/kg (Table 2).

#### Emerging mycotoxins

Boldus, dandelion, devil’s clawroot, ginger, lemon balm, passionflower, valerian, whitethorn and herbal mix for treat insomnia resulted contaminated with emerging mycotoxins (ENNs and BEA) at levels ranging from < LOQ to 137 μg/Kg (Table 2). The higher concentrations were reported for horsetail and *cardus marianus.* In horsetail tablets, ENNs were detected at incidence up to 60% and maximum concentrations comprised between 156.5 and 1188.3 μg/Kg, while BEA was reported in 40% of samples at levels ranging from 25.1 to 52.2 μg/Kg. In *cardus marianus*, ENNs were detected with high incidence (75%) and maximum concentrations between 109.19 and 1378.21 μg/Kg. BEA was detected in 50% of samples at levels ranging between LOQ and 542.7 μg/Kg. Comparing with the information available in bibliography in medicinal herbs dietary tablets, Arroyo-Manzanares et al. (2013) found no mycotoxin presence in a natural extract of *cardus marianus*. Tournas et al. (2012) analyzed AFs presence in 2 samples of alcohol and in 8 samples of oil based *cardus marianus* liquid seed extracts and observed the presence of AFs in 25% of oil based *cardus marianus* liquid seed extracts samples with mean concentration of positive samples of 0.06 μg/kg. Veprikova et al. (2015) reported also high incidence of trichothecens (13–78%), *Alternaria* toxins (22–97%), ZEA (78%) and ENNs (84–91%) in 32 tested samples of *cardus marianus* supplements. ENNs were also detected at maximum concentrations ranging from 2340 to 9260 μg/Kg. In other study, Fenclova et al. (2019) analyzed 26 *cardus marianus* supplements and observed ENNs and BEA presence at incidences between 96 and 100% and maximum concentrations comprising between 798 and 3891 μg/Kg.

#### Zearalenone

ZEA was detected in only one of lemon balm, devil’s clawroot, fucus and ginger samples at concentration of 117, 212.6, 659.7 and 3850.5 μg/Kg, respectively. In boldus, ZEA was reported at high concentrations and incidences, 3 of 4 analyzed samples (75%) resulted contaminated at levels ranging between 1169 and 1995.9 μg/Kg (Table 2). Regarding the information available in literature, Veprikova et al. (2015) reported maximum concentration of 751 μg/Kg in *cardus marianus* supplements, 227 μg/Kg in supplements for treat menopause effects, and 824 μg/Kg in supplements for general health improvement. More recently, Fenclova et al. (2019) reported 89% of 26 *cardus marianus* supplements positives for ZEA at maximum concentration of 282 μg/Kg.

Regarding the studies carried out in the principal medicinal herbs producer countries, in India, Chourasia (1995) studied the presence of AFs, OTA, ZEA and CIT in crude materials and finished herbal drugs. In the crude constituents, AFB1, OTA, ZEA and CIT were detected with incidences of 43, 6, 4 and 6%, respectively, and levels up to 910 μg/kg, while in the finished herbal drugs, only AFB1, OTA and CIT were detected with incidences of 64, 4 and 20%, respectively, and concentrations up to 880, 130 and 150 μg/kg, respectively. In contrast with the present study, AFB1 was reported by these authors with the highest incidence and contents. In China, Zheng et al. (2014) analyzed the presence of AFs, OTA and STG in 244 Chinese medicines. Although the incidences reported by these authors were similar to those obtained in the present study (2–26%), AFB1 was detected in 5.3% of samples at levels up to 1268.6 μg/kg. More recently, also in China, Chen et al. (2020) studied the presence of OTA, CIT and AFs in 48 medicinal herbs, although the major part of samples (81.3%) were positives at levels below the LODs, OTA was detected in the samples at levels up to 515 µg/kg, similar concentration to that obtained in the present work. Concerning the presence of emerging mycotoxins (ENNs and BEA), Hu and Rychlik (2014) reported similar results to those obtained in the present study. These authors analyzed 60 Chinese medicinal herbs and observed that 25% resulted contaminated with one or more of the ENNs and BEA with total levels ranging from 2.5 to 751 μg/kg. Finally, in South African, Areo et al. (2020) analyzed the presence of AFs, OTA and ZEA in 36 South African medicinal plants. These authors observed less concentrations (from 0.03 to 31.46 µg/kg) than those observed in the present study, however mycotoxins were detected with higher incidences. AFs were reported in 86% of samples, OTA in 61% and ZEA in 39%, respectively.

### Comparison between ecological and conventional supplements

The experiment was not designed a priori to evaluate differences between ecological and conventional tablets, so the number of samples analyzed is not equilibrated. The data obtained revealed that 58.3% of ecological samples front 41.1% of conventional samples resulted contaminated by at least one mycotoxin. In ecological samples co-occurrences of two and five mycotoxins were observed front co-occurrences of two, three, four, and five mycotoxins in conventional samples. Regarding mycotoxins contents, significant differences (*p* < 0.05) were observed for OTA, BEA, ENNA_1_ and ENNB between ecological and conventional tablets samples after performing the *t* test, with higher contents observed in ecological samples. Not significant differences were observed for the rest of mycotoxins under this study. Scarce information was available in bibliography comparing mycotoxins contents in ecological and non-ecological medicinal herb samples. However, for other food matrices like cereals, Pleadin et al. (2017) did not find significant differences in mycotoxin contents in 189 samples of unprocessed cereals and 61 samples of cereal-based products originated from conventional and organic production, except of ZEA and FBs.

### Risk assessment

The risk assessment for adult population trough the consumption of medicinal herbs dietary supplements was evaluated. The EDIs obtained for positive samples were compared with the TDIs established.

For ZEA, the EDIs obtained represented a percentage from 0.21 to 11.89% of TDI (Table 3). Boldus tablets were the main contributor to ZEA dietary exposure. OTA, only was present in the group of herbal mix tablets to treat insomnia, and the EDI obtained reached the 3.57% (LB) and 3.92% (UB) of the TWI established.

**Table 3:** Mycotoxin risk assessment through medicinal herbs dietary supplement tablets consumption

Sample	Medium recommended dosage (g)	ZEA			OTA		
		Concentration µg/g	EDI µg/kg bw/day	%TDI	Concentration µg/g	EDI µg/kg bw/day	%TDI
Boldus	1.83	LB^a^					
		1.135	0.0297	11.87	nd	nd	nd
		UB^b^					
		1.138	0.0297	11.89	nd	nd	nd
Ginger	1.49	LB					
		0.962	0.0205	8.19	nd	nd	nd
		UB					
		0.97	0.0206	8.26	nd	nd	nd
Devil’s clawroot	1.16	LB					
		0.053	0.0009	0.35	nd	nd	nd
		LB					
		0.061	0.001	0.4	nd	nd	nd
Lemon balm	0.93	LB					
		0.039	0.0005	0.21	nd	nd	nd
		UB					
		0.0 .00.046	0.046	0.0006	0.24	N nd	nd nd
Fucus	1.3	LB					
		0.131	0.0024	0.98	nd	nd nd	
		UB					
		0.0 .00.046	0.139	0.0026	1.04	nd	nd nd
Herbal mixed for treat insomnia	0.85	LB					
		nd	nd	nd	0.05	0.0006	3.57
		UB					
		nd	nd	nd	0.05	0.0006	3.92

TDI ZEA (0.25 μg/kgbw/day) (EFSA, 2014); TWI OTA (0.12 µg/kg bw/week) (EFSA, 2006)

^a^LB (lower bound);^b^UB (upper bound)

The EDIs obtained for BEA at different scenarios ranged from 0.23 to 3.46% of the TDI established for HT-2 and T-2 and from 0.02 to 0.34% of the TDI established for DON (Table 4). *Cardus marianus* tablets were the main contributor to BEA dietary exposure. The EDIs obtained for the sum of ENNs reached the 0.0048 to 22.2% of the cited TDI, horsetail and *cardus marianus* tablets representing a potential risk, with EDIs that reached 15.6% and 22.2% of TDI, respectively. The percentages obtained decreased to unconcerned (from 0.00048 to 2.2%) when EDIs were compared with the TDI established for DON (Table 4). In general, the consumption of medicinal herbs supplements at the recommended dosage doesn’t suppose a considerable risk even in scarce cases considerable percentages of TDI were reached, highlighting that tablets may constitute an additional source of exposure to mycotoxins.Their control is advisable considering that there is no specific regulation for emerging mycotoxins for food or for food supplements.

**Table 4:** Mycotoxin risk assessment through medicinal herbs dietary supplement tablets consumption

		ENN_S_			BEA		
Sample	Medium recommended dosage (g)	Concentration µg/g	EDI µg/kg bw/ day	%TDI	Concentration µg/g	EDI µg/kg bw/day	%TDI
Valerian	1.4	LB^a^					
		0.057	0.00114	1.15	nd	nd	nd
		UB^b^					
		0.059	0.00118	1.18	nd	nd	nd
Dandelion	1.84	LB					
		0.069	0.00183	1.83	nd	nd	nd
		UB					
		0.072	0.00188	1.88	nd	nd	nd
Boldus	1.83	LB					
		0.0024	6.19e-5	0.062	nd	nd	nd
		UB					
		0.0047	0.00012	0.124	nd	nd	nd
Ginger	1.49	LB					
		0.0046	9.79e-5	0.098	0.0914	0.0019	1.95
		UB					
		0.0067	0.00014	0.14	0.0916	0.002	1.95
Passionflower	1.72	LB					
		0.0046	0.00011	0.11	0.018	0.0004	0.43
		UB					
		0.0068	0.00017	0.17	0.018	0.0004	0.44
Horsetail	2.13	LB					
		0.5114	0.01556	15.56	0.015	0.0005	0.47
		UB					
		0.5128	0.0156	15.60	0.016	0.00049	0.49
*Cardus Marianus*	1.77	LB					
		0.877	0.022	22.18	0.136	0.0034	3.43
		UB					
		0.878	0.022	22.2	0.137	0.0034	3.46
Devil’s clawroot	1.16	LB					
		0.0013	2.1555e5	0.022	nd	nd	nd
		UB					
		0.003	4.9727e5	0.05	nd	nd	nd
Whitethorn	1.36	LB					
		0.017	0.0003	0.34	0.012	0.0002	0.23
		UB					
		0.019	0.0004	0.37	0.013	0.0002	0.24
Lemon balm	0.93	LB					
		0.0022	2.9128e-5	0.029	nd	nd	nd
		UB					
		0.0044	5.7914e-5	0.058	nd	nd	nd
Herbal mixed for treat insomia	0.85	LB					
		0.0004	4.77e-6	0.0048			
		UB					
		0.0025	3.088e-5	0.031	nd	nd	nd

TDI HT2 and T2 (0.1 μg/kg bw/day) (EFSA, 2014)

^a^LB (lower bound);^b^UB (upper bound)

## Conclusion

A simultaneous analysis of 11 mycotoxins (AFs, OTA, ZEA, ENNs and BEA) was performed by LC–MS/MS-IT to investigate medicinal herbs dietary tablets contamination. 36 of 85 analyzed samples resulted positive for at least one mycotoxin. ZEA, OTA and emerging mycotoxins were present in samples with a mean concentration ranging between 65.53 and 1340.11 µg/Kg and incidences from 1 to 34%. ENNB was the most detected mycotoxin. Co-occurrences from two to five mycotoxins were observed in 25% of the samples. Comparing ecological and conventional samples, no significant differences were observed for mycotoxins contents of ZEA, ENNA and ENNB_1_. Data obtained showed that the consumption of this kind of medicinal herbs at the recommended dosage did not increase mycotoxins exposure risk, but vigilance should be kept for high consumers. The rising market of herbal products in Europe and worldwide makes necessary the control of mycotoxins and other chemical contaminates in such products. Poor practices during harvesting, handling, storage, and distribution stages affect the quality and safety of medicinal herbs, so the implementation of good manufacturing practices is essential to reduce mycotoxins presence. Finally, guidelines harmonization in the regulation and control of mycotoxins in medicinal herbs is highly desirable to facilitate the international trade.

## Supplementary Information

Below is the link to the electronic supplementary material.

Supplementary file1 (DOCX 77 kb)

## Abbreviations

ACN: Acetonitrile
AFG2: Aflatoxin G2
AFG1: Aflatoxin G1
AFB2: Aflatoxin B2
AFB1: Aflatoxin B1
AFs: Aflatoxins
BEA: Beauvericin
DON: Deoxynivalenol
EDIs: Estimated daily intakes
ENNA: Enniatin A
ENNA_1_: Enniatin A_1_
ENNB: Enniatin B
ENNB_1_: Enniatin B_1_
ENNs: Enniatins
EU: European Union
FBs: Fumonisins
HMPC: Committee on herbal medicinal products
LC–MS/MS-IT: Liquid Chromatography Tandem Mass Spectrometry Ion Trap
LB: Lower bound scenario
LODs: Limits of detection
LOQs: Limits of quantification
MeOH: Methanol
MEs: Matrix effects
MLs: Maximum limits
QuEChERS: Quick, easy, cheap, effective, rugged, and safe extraction
OTA: Ochratoxin A
SSE: Signal suppression/ enhancement
TDIs: Tolerable daily intakes
TWI: Tolerable weekly intake
UB: Upper bound scenario
ZEA: Zearalenone
