# Supplementary material for: Mycotoxins occurrence in medicinal herbs dietary supplements and exposure assessment
Source: J Food Sci Technol. 2021 Nov 10;59(7):2830–41. doi: 10.1007/s13197-021-05306-y (PMC9207161; doi:10.1007/s13197-021-05306-y)
Supplement: Supplementary file 1 — Supplementary file1 (DOCX 77 kb) [file 13197_2021_5306_MOESM1_ESM.docx]

Table S1. Description of the analyzed samples.

| Main health effect | Assigned number | Ingredients | Tablets recommended dosage (total g) |
| --- | --- | --- | --- |
| Containing one herbal ingredient | | | |
| Control cholesterol, protect liver, detox, weight loss | 1  2 (ecologic)  3  4 | Artichoke (*Cynara scolymus)* | 6-9 (2.4-3.6 g)  2 (1.01 g)  4 (0.84 g)  2-3 (1.2-1.8 g) |
|  | 5 (ecologic)  6  7  8 | Boldus *(Peumus boldus)* | 2 (0.95 g)  4 (2.4 g)  4 (2g)  4 (2g) |
|  | 9  10 (ecologic)  11  12 | Cardus Marianus *(Silybum marianum)* | 3-6 (1.5-3g)  2 (1g)  3 (1.4 g)  3 (1.68 g) |
|  | 13  14  15  16  17 | Dandelion *(Taraxacum officinale)* | 3 (1.5 g)  4 (2 g)  2 (1.38 g)  4-6 (2-3 g)  2-3 (0.9- 1.35) |
| Maintain mobility and joint flexibility | 18  19  20  21 | Devil’s clawroot *(Harpagophytum procumbens)* | 2-3 (1-1.5 g)  2 (1.02 g)  1-2 (0.47-0.98 g)  3 (1.2 g) |
| Anti-inflamatory | 22 (ecologic)  23  24  25 | Ginger *(Zingiber officinale)* | 2 (1.2 g)  4 (1.84 g)  2 (1.12 g)  3 (1.8 g) |
|  | 26 (ecologic)  27  28  29 | Ginkgo *(Ginkgo biloba)* | 2 (0.96 g)  2 (0.9g)  2 (0.75 g)  1-2 (0.47- 0.95 g) |
| Detox, weight loss, reduce blood sugar levels | 30  31 (ecologic)  32  33  34 | Green tea *(Camellia sinensis)* | 6 (2.4 g)  2-3 (0.94-1.41 g)  3 (1.83 g)  3 (1.6 g)  4 (2.62 g) |
|  | 35 (ecologic)  36  37  38 | Red tea *(Aspalathus linearis)* | 2-3 (1.14- 1.71 g)  2-6 (0.7- 2.1)  4 (2 g)  6-9 (3-4.5 g) |
|  | 39 (ecologic)  40  41  42  43 | Fucus *(Fucus vesiculosus)* | 2 (0.55 g)  2 (0.7 g)  1-2 (0.47-0.948 g)  6-9 (3-4.5 g)  4 (1.33 g) |
| Diuretic, improve skin and nails conditions, strengthen bones and tendons | 44  45  46 (ecologic)  47  48 | Horsetail *(Equisetum arvense L.)* | 3-6 (1.5-3 g)  3 (1.8 g)  2 (1.5 g)  3-6 (1.5-3 g)  6 (1.35 g) |
| Reduce anxiety, stress and insomnia | 49  50  51 | Lemon balm (*Melissa officinalis)* | 3 (1.05 g)  2 (0.8 g)  2 (0.94 g) |
|  | 52 (ecologic)  53  54  55 | Passionflower *(Passiflora incarnata L.)* | 2 (1.14 g)  3 (1.11 g)  2-4 (1.12-2.24 g)  4 (2.4 g) |
|  | 56  57  58  59  60 | Valerian *(Valeriana officinalis)* | 3 (1.05 g)  3 (1.05 g)  3 (1.5 g)  4 (1.4 g)  4 (2 g) |
| Regulate blood pressure, regulate heart rate, control cholesterol | 61  62  63  64 | Whitethorn *(Crataegus monogyna*) | 2-3 (0.8-1.2 g)  1-2 (0.47-0.94 g)  3 (0.9 g)  4-6 (1.6-2.4 g) |
| Containing more than one herbal ingredient | | | |
| Treat insomnia | 65  66  67  68  69  70  71  72  73  74  75  76  77  78 (ecologic)  79  80 | Californian tail, californian poppy, hop, whitethorn  Lemon balm, orange flower, valerian  Californian poppy, passionflower, lemon balm, linden, valerian  Californian poppy, passionflower, lemon balm, linden, valerian  Passionflower, hop  Californian poppy, passionflower, lemon balm, linden, valerian  Lemon balm, saffron, melatonin  Whitethorn, passionflower, valerian, rhodiola griffonia  Passionflower, valerian, lemon balm, hop, Californian poppy  Passionflower, Californian poppy, valerian  Passionflower, valerian, eschscholtzia  Lemon balm, passionflower, Californian poppy  Lemon balm, whitethorn, vervain, Californian poppy, linden, chamomile  Lemon balm, orange flower, linden  Passionflower, withethorn, hop, orange flower, valerian  Californian poppy, passionflower, lemon balm, linden, valerian | 2 (1.2 g)  3 (1.2 g)  1 (0.54 g)  1 (0.43 g)  1 (0.54 g)  1 (0.54 g)  1 (0.66 g)  1 (0.61 g)  1 (0.76 g)  1 (0.51 g)  2 (0.86 g)  1 (0.49 g)  1 (0.48 g)  3 (1.92 g)  6 (1.8 g)  2 (1.08 g) |
| Weight loss | 81 (ecologic)  82  83  84  85 | Green tea, moringa, Spirulina, algae, Chorella algae  Artichoke, hop, senna, licorice, rhubarb  Green tea, mate, fucus  Senna, anise, fennel  Fucus, green tea, mate | 4 (1.91 g)  2 (0.97 g)  4 (2.06 g)  2 (0.93 g)  4 (2.06 g) |

**Table S2. Spectrometric parameters of Liquid Chromatography Tandem Mass Spectrometry (LC-MS/MS).**

| Mycotoxin | RetentionTime (min) | DP^a^ | Precursor ion | Quantification ion ^Q^ | | | Confirmation ion ^q^ | | |
| --- | --- | --- | --- | --- | --- | --- | --- | --- | --- |
|  |  |  |  | CE^b^ | Product ion | CXP^c^ | CE | Product ion | CXP |
| AFB_1_ | 9.13 | 46 | 313.1 | 39 | 284.9 | 4 | 41 | 241.0 | 4 |
| AFB_2_ | 9.03 | 81 | 315.1 | 33 | 286.9 | 6 | 39 | 259.0 | 6 |
| AFG_1_ | 8.86 | 76 | 329.0 | 39 | 243.1 | 6 | 29 | 311.1 | 6 |
| AFG_2_ | 9.37 | 61 | 331.1 | 27 | 313.1 | 6 | 39 | 245.1 | 4 |
| ZEA | 10.40 | 26 | 319.0 | 15 | 301.0 | 10 | 19 | 282.9 | 4 |
| OTA | 10.27 | 55 | 404.3 | 97 | 102.1 | 6 | 27 | 239.0 | 6 |
| ENNA | 12.62 | 76 | 699.4 | 35 | 210.1 | 14 | 59 | 228.2 | 16 |
| ENNA_1_ | 12.22 | 66 | 685.4 | 37 | 210.2 | 8 | 59 | 214.2 | 10 |
| ENNB | 11.60 | 51 | 657.3 | 39 | 196.1 | 8 | 59 | 214.0 | 10 |
| ENNB_1_ | 11.89 | 66 | 671.2 | 61 | 214.1 | 10 | 57 | 228.1 | 12 |
| BEA | 12.00 | 116 | 801.2 | 27 | 784.1 | 10 | 39 | 244.1 | 6 |

^a^ DP: decluster potential (volts)

^b^ CE: collision energy (volts)

^c^ CXP: cell exit potential (volts)

**Table S3. Analytical parameters for method optimization.**

| **Mycotoxin** | **Recoveries 100xLOQ ± RSD (%)** | | **Signal Suppression-Enhancer**  **(SSE%)** | **Limits of Detection (LOD) ppb (μg/kg)** | **Limits of Quantification (LOQ) ppb (μg/kg)** |
| --- | --- | --- | --- | --- | --- |
|  | **Intra-day Precision** | **Inter-day Precision** |  |  |  |
| AFB_1_ | 112± 18 | 108± 14 | 58 | 1.5 | 5 |
| AFB_2_ | 76± 20 | 112± 19 | 62 | 0.3 | 1 |
| AFG_1_ | 99± 9 | 108± 20 | 52 | 0.3 | 1 |
| AFG_2_ | 95± 18 | 112± 5 | 46 | 1.5 | 5 |
| ZEA | 109± 5 | 105± 1 | 82 | 3 | 10 |
| OTA | 119± 1 | 115± 5 | 79 | 1.5 | 5 |
| ENNA | 98± 1 | 117± 20 | 94 | 0.3 | 1 |
| ENNA_1_ | 87± 18 | 116± 3 | 74 | 0.15 | 0.5 |
| ENNB | 73± 20 | 111± 5 | 83 | 0.15 | 0.5 |
| ENNB_1_ | 109± 9 | 103± 1 | 89 | 0.3 | 1 |
| BEA | 103± 9 | 105± 12 | 98 | 0.3 | 1 |

Figure S1. Incidence of mycotoxins (%) detected in analyzed tablets (n=85).


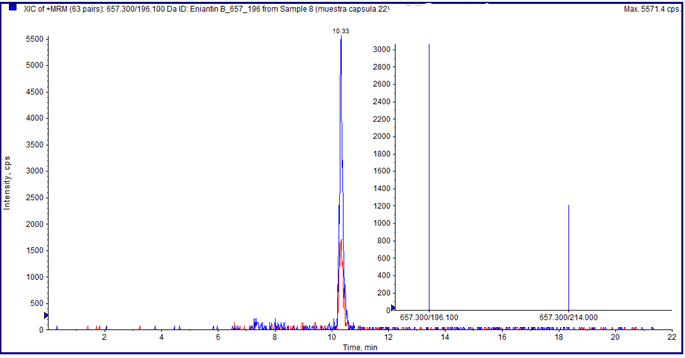


**Figure S2. Chromatogram obtained from a sample of whitethorn naturally contaminated by ENNB (12.25 μg/kg) Retention Time= 10.33 min.**
